# *Mariner* transposons are sailing in the genome of the blood-sucking bug *Rhodnius prolixus*

**DOI:** 10.1186/s12864-015-2060-9

**Published:** 2015-12-15

**Authors:** Jonathan Filée, Jacques-Deric Rouault, Myriam Harry, Aurélie Hua-Van

**Affiliations:** Laboratoire Evolution, Génome, Comportement, Ecologie UMR9191 CNRS, IRD Université Paris-Sud, Gif-sur-Yvette, France; UFR de Sciences, Université Paris Sud, Orsay, France

**Keywords:** *Rhodnius*, Transposable element (TE), Miniature inverted repeat transposable element (MITE), *Mariner*, Horizontal transfer

## Abstract

**Background:**

The Triatomine bug *Rhodnius prolixus* is a vector of *Trypanosoma cruzi*, which causes the Chagas disease in Latin America. *R. prolixus* can also transfer transposable elements horizontally across a wide range of species. We have taken advantage of the availability of the 700 Mbp complete genome sequence of *R. prolixus* to study the dynamics of invasion and persistence of transposable elements in this species.

**Results:**

Using both library-based and *de novo* methods of transposon detection, we found less than 6 % of transposable elements in the *R. prolixus* genome, a relatively low percentage compared to other insect genomes with a similar genome size. DNA transposons are surprisingly abundant and elements belonging to the *mariner* family are by far the most preponderant components of the mobile part of this genome with 11,015 m*ariner* transposons that could be clustered in 89 groups (75 % of the mobilome). Our analysis allowed the detection of a new *mariner* clade in the *R. prolixus* genome, that we called *nosferatis*. We demonstrated that a large diversity of m*ariner* elements invaded the genome and expanded successfully over time *via* three main processes. (i) several families experienced recent and massive expansion, for example an explosive burst of a single *mariner* family led to the generation of more than 8000 copies. These recent expansion events explain the unusual prevalence of *mariner* transposons in the *R. prolixus* genome. Other families expanded *via* older bursts of transposition demonstrating the long lasting permissibility of *mariner* transposons in the *R. prolixus* genome. (ii) Many non-autonomous families generated by internal deletions were also identified. Interestingly, two non autonomous families were generated by atypical recombinations (5' part replacement with 3' part). (iii) at least 10 cases of horizontal transfers were found, supporting the idea that host/vector relationships played a pivotal role in the transmission and subsequent persistence of transposable elements in this genome.

**Conclusion:**

These data provide a new insight into the evolution of transposons in the genomes of hematophagous insects and bring additional evidences that lateral exchanges of mobile genetics elements occur frequently in the *R. prolixus* genome.

**Electronic supplementary material:**

The online version of this article (doi:10.1186/s12864-015-2060-9) contains supplementary material, which is available to authorized users.

## Background

The Triatominae blood-sucking bugs (Hemiptera, Reduviidae, Triatominae) are vectors of *Trypanosoma cruzi* (Kinetoplastida, Trypanosomatidae), the ethiologic agent of Chagas disease. Chagas disease is the most important parasitic disease in Latin America with 7 to 8 million affected people and is one of the most neglected diseases in the world (WHO, 2014). To date, about 140 species of Triatominae have been described into three main genera: *Rhodnius, Triatoma,* and *Panstrongylus*. Recently, the genome of *R. prolixus* has been sequenced (www.vectorbase.org). The availability of high throughput sequencing data has refined our understanding of functional genomics and gene expression and also the identification of adaptation mechanisms that may involve structural variations including gene duplication or transposition of mobile elements [1]. In addition *R. prolixus* are suspected to transmit transposable elements (TE) horizontally across phyla [2]. TEs, which represent an important part of eukaryotic genomes, play important roles in genome size, genome adaptability, and genome structure and functions [3, 4]. At the gene level, they can trigger dramatic gene inactivation or temperate regulation changes. TEs are usually silent but can occasionally reactivate under environmental changes, notably through epigenetic changes affecting TE copies [5–7]. Hence this reactivation may lead to transposition burst, which will increase (through transposition or recombination) adaptability, genetic diversity, and probability to create beneficial/adaptive alleles [8]. However, TEs have to undergo frequent horizontal transfers (HTs) between different species to avoid stochastic losses [9]. A growing number of cases of TEs HT have been reported in the literature but their underlying mechanisms are still unknown [9]. It has been shown that four TE families in the genome of *R. prolixus* are almost identical to mammalian TEs [2]. These data support the existence of recent HTs of diverse TEs between this species and their mammalians hosts. They may also indicate that this haematophagous bug plays a pivotal role in the transmission of TEs across a wide range of species. Recently, six additional MITEs almost identical between *R. prolixus* and the silkworm *Bombyx mori* have been evidenced [10]. Taken together these data suggest that *R. prolixus* is an interesting model to document the evolutionary dynamics of TEs, notably the role played by the host/parasite interactions in the mechanism of HT events of transposons.

In this paper we explored the complete genome of *R. prolixus* for transposons and their non-autonomous derivatives using a combination of library-based and *de novo* methods. We found that TE derived sequences compose 5.8 % of the *Rhodnius* genome, a relatively modest contribution in comparison to other insect genomes. But DNA transposons are surprisingly abundant and especially a very large diversity of *mariner* families accounts for two third of these TEs. We demonstrate that the dominance of *mariner*-like transposons is the result of recent and older burst events in addition to more continuous expansion of other families. The ongoing invasion of *mariner* elements is also associated with multiple generations of non-autonomous derivatives that have subsequently expanded. Finally, the identification of several HTs sharing with various species suggests the existence of horizontal transfers of TEs which participated to the recurrent invasion of the *R. prolixus* genome by exogenous *mariner* transposons.

## Methods

### Data collection and availability

*Rhodnius prolixus* assembled genomic sequences (RproC1) were downloaded from VectorBase (htps://www.vectorbase.org/organisms/rhodnius-prolixus). We analyzed TEs in the whole genome using RepeatMasker with default parameters (http://www.repeatmasker.org) and a library of Metazoan TEs extracted from Repbase (http://www.girinst.org/repbase/)[11].

Python scripts and raw data including TE sequences, consensus, alignments and phylogenetic trees… are available at: http://echange.legs.cnrs-gif.fr:5000/fbsharing/LUGs8EBq

### Library based method for Tc1-mariner Element searches

TBLASTN searches for *Tc1-mariner* elements [12] was run on the *R. prolixus* genome, using 8 *mariner* transposase protein sequences, representative of the major subfamilies and 15 non-*mariner* transposases (Additional file 1: Table S1) . We obtained 51,271 and 2711 hits respectively. A suite of python scripts was then used for:

i) Reconstitution of copies by associating hit distant of less than 1000 bp, in correct orientation
ii) Filtering out any copies less than 400 bp-long
iii) Extraction of all the sequences with or without 500 bp flanking sequences each side to get full copies
iv) Clustering copies (without flanking sequences) with Usearch (−id 0.8, −rev) [13]
v) Aligning sequences (with flanking sequences) in each cluster with MAFFT [14] and refined by hand using AliView [15] allowing to identify the complete sequences
vi) Filtering out sequences with “N”, assembly-truncated copies, and duplicated copies (resulting from segmental duplication and not from transposition, as determined by the flanking sequences.
vii) Trimming flanking sequences and generating nucleotide consensus (majority rule with keeping the longest elements), then protein consensus

### De novo identification of MITEs

We used a suite of python scripts gathered under the name AutoMitaur (Hua-Van, unpublished) and available at http://www.egce.cnrs-gif.fr/wp-content/uploads/2014/04/AutomitAur.v1.0.1.zip.

Briefly, in this suite of script, BLASTN is used to compare a genome against itself for short hits at least 11 bp-long, distant of 750 bp at most, and in inverted orientation (TIRs). The TIRs, the intervening sequence plus 60 bp flanking sequences on each side are then extracted. Sequences are then clustered and copies with similar flanking sequences are removed. Several filters are applied and only groups with at least ten independent sequences that reach a certain level of homogeneity between the sequences and display *bona fide* TIRs are kept. A consensus sequence is then determined for each cluster. The pipeline also includes a step consisting of searching (BLASTN-SHORT) for putative autonomous partners, by using the defined TIR sequences as queries against the input genome, keeping only sequences larger than 1 kb. The putative longer elements are then searched against the RepBase protein database (31/01/2014 version) using BLASTX, to automatically identify the potential associated super-family. In parallel, a BLASTX search was realized with the MITE consensus sequences as a query, against the database.

Out of a raw output of 107 clusters, we could then select 41 MITE clusters for further analysis.

### TE Classification and phylogenetic analyses

We classified clusters of the *Tc1-mariner-IS630* super-family to define homogeneous groups. This computation is based on the UPGM-VM method, an ascending hierarchical classification analogous to the classical UPGMA, with two main differences: 1) there is no arithmetical mean, the sequences are aligned two-per-two and the corresponding distances are computed; 2) the metric varies with the ascending classification. At the beginning, an alignment gap is considered as a fifth nucleotide, and its weight is progressively and rapidly set to zero. This variation of the metric allows gathering in the same group a complete sequence and the corresponding truncated or deleted sequences such as MITEs [16].

*R. prolixus* elements found in this study were added to a set of 309 complete sequences previously published in GenBank and representatives of the main clades of the Tc1-m*ariner*-IS630 SuperFamily : *mariner* (Briggsae, Cecropia, Elegans, Irritans, Mellifera, Mauritiana, Vertumnana), maT (mori), Tc1, Tc2, Tc3, Tc4, Tc5, Tc6, Gambol, Pogo, Fot, Lemi, Plant *mariner*, Impala, IS630, IS870. We added the 36 *Drosophila* sequences described by Wallau et al. [17] and the consensus sequences found here in *R. prolixus*.

For the phylogenetic analysis we used a representative set of *mariner* transposase from Repbase covering all the known clades or lineages of the super-family [11, 16, 18]. Sequences were aligned using MUSCLE with default parameters and conserved parts of the alignments usable for phylogenetic analyses were chosen using Gblocks [18, 19]. The best-fitting ML model was selected using Protest and the tree was computed using PhyML 3.0 [20]. Branch supports were calculated using a LRT Shimodaira-Hasegawa (SH) procedure.

### HT identification

We compared *R. prolixus mariner* consensus sequences to Genbank and WGS NCBI databases (ftp://ftp.ncbi.nlm.nih.gov/) using BLASTN searches [12]. Candidate elements for HT were identified as sequences with more than 75 % of nucleotide identity over more than 90 % of the query sequences. To discard potential cases of contamination with foreign DNA, each genomic context of the putative elements was carefully examined: each 50 kbp adjacent segment was inspected with a BLASTN procedure and only elements within a conserved synteny block were conserved. Cases of HTs were then validated using phylogenetic analyses.

### TE amplification dynamics

We inferred species-specific amplification dynamics of single lineages using a new method based on the phylogenetic tree node distributions over time. This method relies on the topology of the phylogenetic tree and offers a visualization of the variation in transposition rate per copy over time. More details are available in Le Rouzic et al. [21].

## Results and discussion

### Tc1-mariner elements dominate the mobilome of R. prolixus

**Fig. 1:**
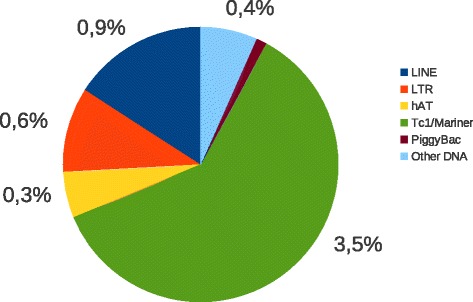
Repartition by super-families of the *R. prolixus* mobilome. Numbers indicate the percentage of the genome occupied by each super-family

We explored the complete 700 Mb genome of *R. prolixus* for TEs using a RepeatMasker/RepBase strategy (see methods), and found a total of 40.9 MB of repeated sequences representing 5.8 % of the genome. TEs abundance in the *R. prolixus* genome is relatively low compared to other insect genomes with similar genome size. For example TEs constitute 40 % of the 530 Mb genome of the silkworm *Bombyx mori* [22]*.* Although there is a positive correlation between the genome size and the abundance of TEs [23], insects with smaller genomes such as *Drosophila species* (110–180 Mb), the beetle *Tribolum castaneum* (152 Mb)*,* the honeybee *Apis mellifera* (236 Mb) or the mosquito *Anopheles gambiae* (250 Mb) display total TE contents generally equivalent or higher (respectively 2.7 % to 25, 6, 5.9 % and 16 %)[24–27]. Moreover, the repartition between the main classes of transposable elements is fundamentally different in *R. prolixus* when compared to other insects (Fig. 1). Indeed, the *R. prolixus* mobilome (all the mobile elements in a given genome) is largely dominated by DNA transposons that represent 75 % of the mobilome, whereas in *B. mori*, *Drosophila* species, *T. castaneum* and *A. gambiae,* retrotransposons and their derivatives are considerably more prevalent (respectively 89 %, 67 % to 93 %, 87 % and 72 %) [24–27]. An additional striking feature of the mobilome of *R. prolixus* is the preponderance of elements from the *Tc1-mariner-IS630* super-family (Fig. 1). On its own, the *Tc1-mariner-IS630* superfamily represents around two third of the mobilome. The other superfamilies of DNA transposons (*hAT*, *piggyBac*, Tourist, Transib…) play an anecdotal role in the representativeness of class II TE in this genome.

### Large diversity of mariner elements in the R. prolixus genome

In order to identify the different *Tc1-mariner* transposable elements, we used a homology-based approach (TBLASTN), starting with two sets of transposases, one composed of eight *mariner* transposases representing the major *mariner* subfamilies [16, 17], the other set comprising fifteen transposases belonging to other Tc1-like families (classified according to the catalytic domain as in [28]) (Additional file 1: Table S1). The *mariner* search retrieved a total of 11,015 copies that could be clustered in 89 groups of copies with similarities higher than 80 % and that likely represent functional lineages (i.e., copies within one lineage can cross-mobilize copies from the same lineage, due to high sequence similarity, usually over 80 %). On the opposite, the non-*mariner* search retrieved only 502 copies, clustered in 52 groups (Additional file 1: Table S1). This revealed that the large domination of the *Tc1*-*mariner-IS630* elements in *R. prolixu*s is mainly due to elements of the *mariner* family (characterized by a DD(34)D catalytic domain) both at the abundance and the diversity levels and we subsequently focused on this family.

Most of the 89 *mariner* clusters comprised less than 5 copies, and we only retained for subsequent analysis a set of 32 lineages with at least 5 independent copies, representing a total of 10,836 total copies (from which 5011 appeared independent and not truncated by the assembly, i.e. with no “N”) (Table 1). For most of the lineages, we could retrieve the Terminal Inverted Repeats (TIRs) necessary for transposition as well as the target site duplication (TSD). As expected, all the complete copies were bordered by a TA dinucleotide TSD, and the TIR sequences presented high similarities for lineages of the same subfamily. This is expected because lineages from the same subfamily share a more recent common ancestor than lineages from different subfamilies [17]. Additionally, the TIRs directly interact with the transposase, and coevolution is then expected to occur [29]. On the opposite, the TIR sequences may be quite different between most distantly-related subfamilies (Table 1).

**Table 1:** Characteristics of *mariner* lineages identified in the *Rhodnius prolixus* genome. Column “Clean Independent Copy Number” reports the number of copies not truncated by “N” and corresponding to true transposition events (different flanking sequences). Column “Potentially Active Copies” indicates if at least one complete ORF (>1000 bp) has been found among copies

Clusters	Total Copy Number	Clean Independent Copy Number	Subfamily	Lenght	TIR lenght	TIR sequences	Potentially Active Copies	Remarks
Rpmar10	125	93	*vertumnana*	1305	35	CGAGGGGCACTACTTATATTTTGAGCCTTGGCAAC	Yes	Putative Horizontal Transfer
Rpmar16	6	5	New	1320	28	CGAGGGTCATTCGTAAAGTAAGGTTCCC	Yes	
Rpmar9	91	52	New	1050	33	CGAGGGTCATTCAATAAGTAACGAGACAAATTA	No	
Rpmar13	6	3	New	1312	30	CGAGGGTGAATCAAATATAAACGAGACTT	No	
Rpmar33	8	7	New	1375	27	CGAGGCATGTCCAGAAAGTAAGTGTA	No	
Rpmar48	48	46	New	876	29	CGAGGGTTGGCTGAAAAGTAATGCACACA	Deleted	
Rpmar0	8041	3259	*irritans*	1291	28	CGAGGGTCGTTTGAAAAGTCCGTGCAAA	Yes	Putative Horizontal Transfer
Rpmar35	195	26	*irritans*	898-917	28	CGAGGGTCGTTTGAAAAGTCCGTGCAAA	Deleted (Rpmar0)	
Rpmar22	37	30	*irritans*	1270	28	CGAGGGTGGTTTGAAAAGTTCTCGGAAT	Yes	
Rpmar5	32	24	*irritans*	1285	33	ACACATGGGCTGAAAAGTCCCGGGCCTAACACA	No	Putative Horizontal Transfer
Rpmar1	767	401	*drosophila*	1315	29	CGAGGTGTGTTCAAAAAGTAACGGGAATT	Yes	
Rpmar6	488	246	*drosophila*	1329	29	CGAGGGGGTACCCAAAAATAACCGGAATT	Yes	
Rpmar26	9	8	*drosophila*	1320	29	CAGGGTGTGTATTTTAAGTAATGAGAATA	No	
Rpmar17	205	140	*drosophila*	922	57	CGAGGTCTGTAAATTAAGTAATGAGACTGATTTTTTTAATTTTTTTTATTCAAAAAG	Deleted/Recombined	
Rpmar63	154	135	*drosophila*	897	27	CGAGATTTGGTTATTAAATAACGAGAC	Deleted	Putative Horizontal Transfer
Rpmar11	73	56	*drosophila*	921	33	CGAGGTATGTTCAAAAAATAAGGTGAATTTTCA	Deleted	
Rpmar83	20	17	*drosophila*	826	32	CGAGGTATGGCTATTAAATAACGAGACTGATG	Deleted	
Rpmar57	17	14	*drosophila*	918	32	CGAGGTCTGTTCAAAAAGTATCACGAATTTTG	Deleted	Putative Horizontal Transfer
Rpmar65	5	5	*drosophila*	898	28	CAGGGTGCGTTCCAAAAGTAATGCAATT	Deleted	
Rpmar4	165	132	*mellifera*	1333	31	WYGGGTTGGCCAATAAGTTCGTTCGGTTTTT	No	
Rpmar12	40	29	*mellifera*	1296	31	WTGGGTTGGCAACTAAGTCATTGCGGATTTT	No	Putative Horizontal Transfer
Rpmar23	25	20	*mellifera*	1291	30	TTGGGTTGGCAACTAAGTAATTTCGGTTTT	No	
Rpmar27	22	19	*mellifera*	1251	33	TAATGGGTTGGGGAAAAATAAATCCATTATTTT	No	Putative Horizontal Transfer
Rpmar20	6	6	*mellifera*	1285	30	TCGGGTTGGCAAATAAGTCCTTTCGATTTT	No	
Rpmar15	18	15	*mauritiana*	1281	29	CaaAGGTGCATAAGTTTTTTCCGGTTTAA	Yes	
Rpmar19	16	8	*mauritiana*	1291	30	TCGGGTGTGTGCATTAATTTTAAGGATTTT	Yes	
Rpmar21	7	7	*mauritiana*	1299	30	CATAGGTGTAGAAGTATGAAACCGGAATTT	No	
Rpmar14	148	125	*cecropia*	1293	22	TTGGGTTATCCAGAATATAATG	No	
Rpmar24	28	16	*cecropia*	1295	31	TTGGGTTGGTGCAAAAATAATGCAGGTTTTT	Yes	
Rpmar31	14	29	*cecropia*	908	31	TYGGGTTGTCAAGTATGAATGGAGCAAAGTT	Deleted	
Rpmar49	13	13	*capitata (?)*	970	221	WTAGGGGGACCGAAAAGTAATCAAAA…	Deleted/Recombined	
Rpmar30	7	6	*capitata (?)*	1258	25	ATRGGGGCACCGGAAAGTAATGTTT	No	Putative Horizontal Transfer

The initial 11,015 sequences, consisting only of sequences exhibiting homology with transposase sequences, covered about 7 Mb of the genome, mainly due to the 32 lineages. By comparison the 503 non-*mariner Tc1*-like copies covered only 0.35 Mb. However, when the full nucleotide consensuses derived from the 32 *mariner* lineages were used as seeds in a RepeatMasker search, 26.4 Mb were masked, slightly more than the initial search using RepBase as the seed library (24.5 Mb). Then, our TBLASTN methodology based on transposases is not fully exhaustive since it did not allow the recovery of all *mariner* sequences including degenerated or highly divergent copies. The most probable explanation is that a large amount of *mariner* fragments, lacking ORF sequences, or shorter than 400 bp (our filtering threshold) exist in the *R. prolixus* genome. For example, the Rpmar63 encompasses 153 identifiable sequences with our pipeline (Table 1) but a BLASTN with the consensus sequence identify 580 additional short and fragmented sequences. Another problem is the level of assembly quality of the genome. Indeed, the 55,000 contigs include a large proportion of small contigs (only 13 % of them are bigger than 10,000 bp). That may prevent the recovery of long-enough copies, and ultimately makes impossible a precise estimation of the amount of repeated sequences (which often corresponds to unmapped small contigs). Nevertheless, and although both methods are homology-based, our TBLASTN-based method appears more efficient than the RepeatMasker/RepBase strategy, that likely underestimates the amount of repeated sequences, probably due to high divergence between the sequences in the library and the elements in the genome.

Besides these methodological limitations, two facts still account for the exceptional situation encountered in the *R. prolixus* genome regarding the *mariner* elements. The first is that the huge amount of *mariner* sequences is mainly due to one single lineage (Rpmar0) comprising more than 8000 copies (73 % of all *mariner* elements). Furthermore, seven other lineages display more than 100 copies. *Mariner* is described as a low copy number family, although high copy number lineages have occasionally been described in some species (see for example [30]). In a recent analysis of 20 *Drosophila* genomes [17] the most prolific *mariner* lineage exhibited about 500 copies in one genome, most of the other consisting of less than 50 copies/per genome and usually less than 10. The *R. prolixus* genome appears then rather permissive for *mariner* amplification, for reasons that still remain to be deciphered.

The second peculiarity in this genome is the huge diversity of *mariner* elements. 89 different clusters (suggesting about the same number of functional lineages) have been identified. Even by considering only those with at least 5 copies, it is still more than 30 different lineages coexisting in the very same genome, just a few less than in the recently analyzed 20 *Drosophila* genomes, taken as a whole. Indeed, no more of 23 lineages > 5 independent copies have been identified within one single *Drosophila* genome [17]. The *R. prolixus* genome then appears so far the most comfortable ecological niche for *mariner* elements.

All these lineages fully covered the known *mariner* diversity and possibly formed at least one new subfamily. We first performed a classification of the *R. prolixus* nucleotide sequences using a clustering method (UPGM-VM) based on the whole nucleic sequences of 309 Tc1-*mariner* sequences. This classification allows the use of a large dataset in a reasonable calculation time, including distantly related Tc1 and Tc3 sequences found in animals, plants, fungi and bacteria (Additional file 2: Figure S1). The resulting classification revealed the clustering of *R. prolixus* sequences within known clades/subfamilies with the exception of four lineages that may define a new subfamily called *nosferatis* (*Nos* in Additional file 2: Figure S1).

**Fig. 2:**
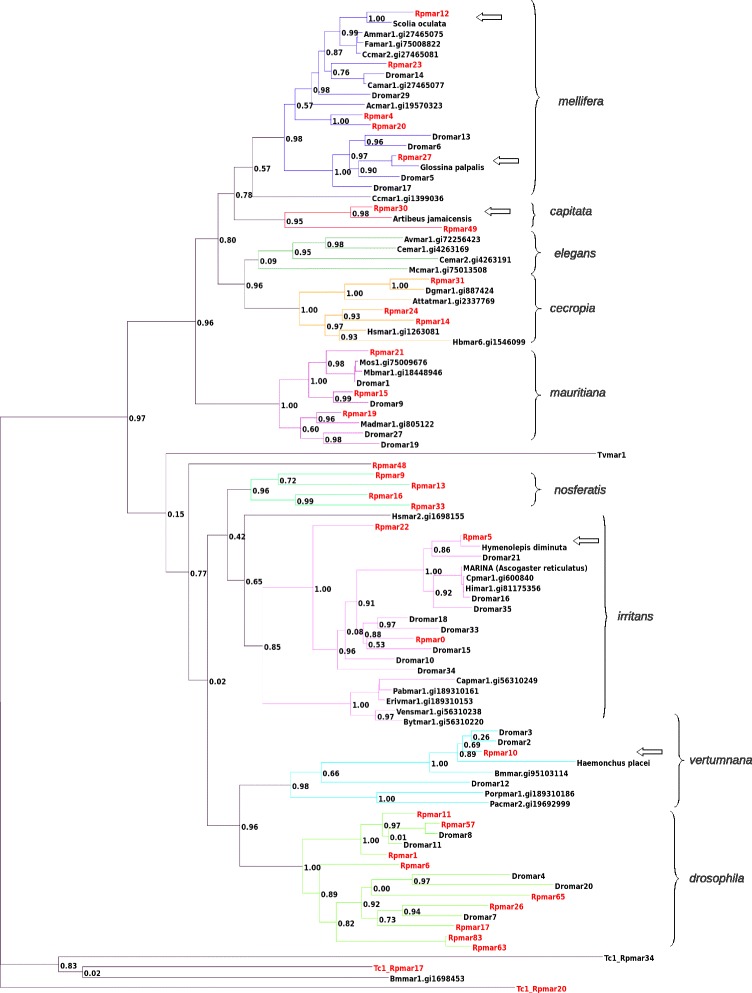
ML phylogeny of the *Tc1-mariner* superfamilies. *R. prolixus* sequences are framed in red, arrows represent the putative cases of HTs and the numbers beside each node indicated the value of the SH-like statistical test. Brackets and branches with the same colors represent the traditionally recognized subfamilies of the *mariner* elements

To confirm these first results, we performed a ML phylogenetic analysis of the translated consensus of 32 lineages plus representative transposase sequences of each *mariner* subfamily (Fig. 2) Again, *R. prolixus mariner* lineages were found in almost all recognized subfamilies. Only the scarce subfamily *elegans,* and the *bytmar*-like clade of the large *irritans* subfamily have no representatives in the *R. prolixus* genome. We could also confirm that some lineages are not included in the known subfamilies and may represent the new never-described subfamily *nosferatis.*

The typical *mariner* size is between 1280 and 1350 bp, which is supported by the size of most of the consensus sequences reported in Table 1. Among the 32 lineages analyzed, we found only 9 lineages with at least one full-length copy with an uninterrupted ORF that could witness recent potential activity. Furthermore, we could identify ten lineages for which the consensus sequence (constructed in a way to fit the most complete element) is between 800 bp and 1000 bp-long, meaning that these lineages are only made of shorter elements and then obviously represent non-autonomous lineages. It is noteworthy that six of these lineages belong to the subfamily *drosophila*, already known to easily generate such kind of deleted lineages in the 20 Drosophila genomes [17]).

Disregarding the fact that these lineages have kept a reasonable size, they could represent lineages on the way of becoming MITEs (Miniature Inverted-repeat Transposable Elements), that amplify using the transposase of other closely related lineages that share almost identical TIR sequences. MITEs are usually present in high copy number, and supposed to derive from full-length lineages by successive shortening of the internal part, combined with elevated sequence degeneracy, and in some cases rearrangement, while keeping the ability to be mobilized [31].

One example of ongoing “MITEzation” is provided when comparing one of these shorter lineages (Rpmar35), which is actually directly derived from the dominant Rpmar0 lineage by internal deletion. Yet, Rpmar35 is mainly composed of 2 sets of shorter sequences similar to Rpmar0, and having obviously transposed after internal deletion in the transposase sequence of a Rpmar0 copy.

**Fig. 3:**
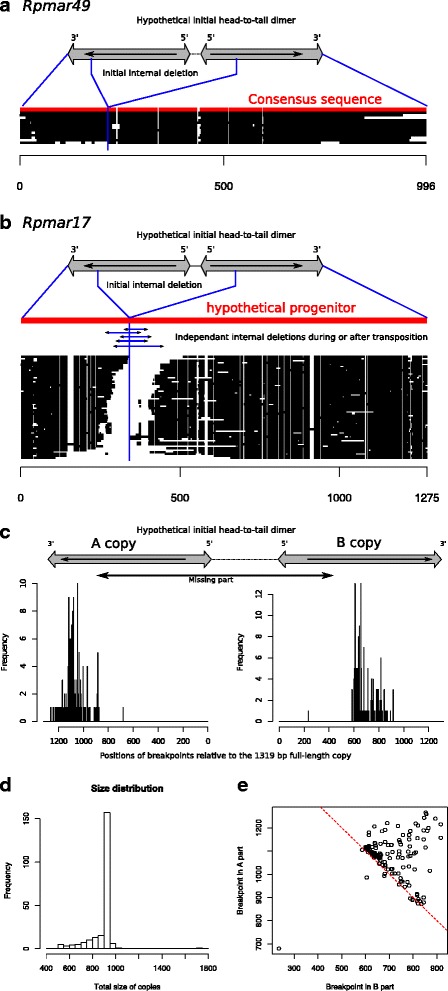
Analysis of shorter *mariner* lineages made of rearranged sequences. **a** and **b** For the two examples presented here, copies where aligned and the hypothetical initial structure is shown above. The putative initial deletion event is shown in blue. For Rpmar17, the vertical blue line indicates the limit between the two non-overlapping regions, and blue horizontal arrows reflected the further internal deletions that could have take place after or during the amplification process. **c** Histograms showing the positions of the breakpoints in copies with a rearranged structure. The numbering is after the full-length non-rearranged copy (1319 bp). Copies were retrieved with megablastn using the full-length sequence as query, and were reversed-blasted against the full-length sequence. Only copies displaying hits on both plus and minus strands, and with no other deletions, were kept (227 copies). **d** Size distribution of the copies. **e** Scatter plot showing breakpoints in A part versus B part for each copy. The red dotted line represents a size limit of 938 bp

Other notable short lineages include Rpmar49 and Rpmar17 that both present exceptionally long TIRs. For Rpmar49, it is visible that this lineage resulted from the replacement of the 5’ part of the element by a 221 bp-long sequences corresponding to the 3’ part, explaining the long TIRs (Fig. 3a). This rearrangement is confirmed by the presence of transposase homology in the rearranged part. The 13 independent sequences (i.e. having amplified by true transposition) are quite homogeneous in size and sequences, providing evidence that these non-autonomous sequences all derived from a unique progenitor that has parasitized an autonomous element to amplify. The simplest hypothesis is that an initial deletion occurred between two head-to-tail copies (with or without intervening sequences) by an abortive gap repair process that is known to be responsible for internal deletion of TEs [32].

All the Rpmar17 sequences (except two that correspond to near full-length copies) seemed to have experienced the same kind of rearrangement (5’ part replacement with 3’ part), as for Rpmar49. A striking difference is however that few copies exhibit identical recombination breakpoints, as shown from a subset of complete sequences that we could easily align (Fig. 3b). All the breakpoints seem however localized in the same region. This case is at first glance puzzling, but can actually gives insights on possible initial events responsible for this lineage. One hypothesis is that all these different sequences, made of a transposed 3’ part having replaced the 5’ part, could result from an initial head-to-tail *mariner* dimer or close copies. From it, a shorter element would have arisen by internal deletion, leaving only extremities made of 3’ parts. After (or during) amplification of this progenitor sequence, resulting copies would have suffered new independent deletions all localized around the hypothetical initial breakpoint. This hypothesis suggests then two unrelated process (the first rearrangement/deletion event and the subsequent deletions centered around the breakpoints.

We performed an additional analysis relying only on the position of the breakpoints relative to the non-rearranged full-length copy, avoiding the problematic step of aligning. A similar pattern was observed for 227 independent rearranged copies, including the variable position of the breakpoints (Fig. 3c).

We noticed that the longest copy that could represent the initial deleted progenitor is more than 1700 bp long. Curiously all the other rearranged copies but one are less than 1000 bp long, with the majority between 900–950 bp (Fig. 3d and e). Element size, as well as internal structure, can influence the transposition efficiency [31, 33, 34]. However, in our case, successful transposition is not observed since most copies exhibit different breakpoints: they are probably not derived from each other by transposition. Hence the size homogeneity is not the result of selection for transposition ability and the observed necessity for a certain size range is difficult to understand here. Alternatively, the apparent propensity to obtain 950 bp copies after deletion could result from structural particularities in the breakpoints regions. For example, these regions could be hotspots for double strand breaks repair [35], or prone to be joined together during abortive gap repair. Indeed, it was already shown that deletions are not totally random in transposable elements and may depends on sequence characteristics [35].

### R. prolixus mariner elements generate a limited set of MITEs smaller than 900 bp

These few examples described before illustrate the fact that *mariner* transposons can generate shorter lineages that are able to amplify, although no lineages shorter than 900 bp could be identified. Since MITEs are usually shorter and are sometimes related to autonomous elements only by very short sequences corresponding to TIRs with or without subterminal sequences, they can totally lack any similarities with coding sequences (ORF), and then cannot been retrieved with our method [31]. In order to complete the *mariner* landscape, we then used a *de novo* approach based on the presence of short inverted repeats less than 750 bp apart; we retrieved 107 clusters of potential MITEs with at least 10 copies. 33 of them were found to be potentially inserted in a TA TSD, among which the six more abundant (more than 100 copies). For each cluster, a search for longer elements bordered by the same TIRs was run and longer copies blasted against the protein repbase to detect homology with transposase. The same was also carried out using representative or consensus sequences of the different lineages. We then selected 41 clusters meeting one of these criteria (TSD with TA, or Tc1-*mariner* transposase homology), for further analysis and manual inspection. However, very few families could be confirmed to be *Tc1-mariner* MITEs. Indeed, for some of them, no similarity to *Tc1-mariner* sequences could be found, in internal part or within the TIRs. For some other elements the TSD was determined to be larger than just the typical TA observed for *Tc1-mariner*, suggesting these elements could belong to other super-families (CACTA, P, *hAT*, *piggyBac*…). For ten MITE lineages, no clear TSD and TIRs could be defined, weak homology often extending outside the putative limits of the elements. Finally, for several MITE lineages, the homology found in longer element sharing the same TIRs was due to the nested insertion of a *Tc1-mariner* element in a non- *Tc1-mariner* MITE.

Among the fifteen clusters that may ultimately belong to *Tc1-mariner* (Table 2) a longer potential partner copy could be identified in only four cases. In all other cases, the superfamily is deduced from similarity in the TIRs. Some of these lineages are described below in more details.

**Table 2:** List of MITE clusters that belong to the *Tc1-mariner* superfamily. Only clusters with at least one sublineage may represent bona fide MITEs. (a) independent copy number (b) minimum and maximum size are given

Clusters	Copy number (a)	Sub lineages	Confirmed family	Partner in *Rhodnius* genome	Size (b)	TSD and TIRs	Remarks
MITE_9	118	No	*mariner*	Rpmar0	299-845	TACGAGGGTCGTTTGAAAAGTCCGTG	Internal deletions
MITE_95	16	3	*mariner*	Rpmar10	482-803	TACGAGGGGCACTATTTATATTTTGAG	
MITE_147/170	11	No	*mariner*	Rpmar63	172-743	TACGAGGTGTGGCTATTAAATAACGAGACT	Internal deletions
MITE_100	68	1	*Tc1-like*	No	560	TACACTGATGGACAAAATTAACGCACCACC	
MITE_109	37	3	*Tigger*-like	No	300-647	TACAGTGGTACCTCGGTTTTCGAA	
MITE_120	174	3	*Tigger*-like	No	282-649	TACAGTGGAGTCTCGGTTATCCGT	
MITE_51	70	2	*Tigger*-like	No	307-796	TACAGTACAACCTCGAT	
MITE_125	28	3	*Tigger-*like	No	402-743	TACAGTAGACTCTCAGAAATCCGG	
MITE_185	11	3	*Tigger*-like	No	274-312	TACAGTAAGACCCCGCTTAACGCG	Putative HT
MITE_113	11	1	*Tc1*-like	No	522-556	TACAGGGGGTGGACAAAAAAATGGAAACAC	
MITE_83.0	11	?	*Tc1*-like ?	No	489-1073	TACAGGGTGACCAGAGTTATATGCTCCACCCACTTTTTT	
MITE_260.1	38	2	IS*630*	Tc1_Rpmar24	91	TATAGCCAAGCGACA	Prokaryote

**Fig. 4:**
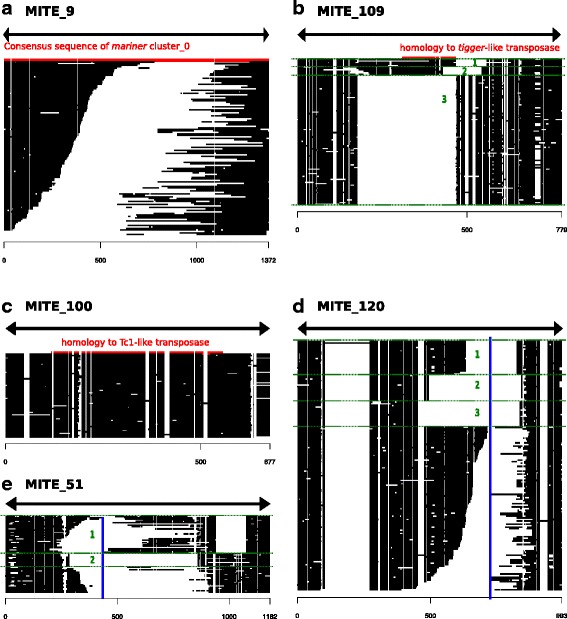
Sequence alignments of different MITE lineages (**a**-**e**) with longer autonomous partner or with highlighted homology region to transposase sequence. For each alignment, sequences are in *black*, showing gaps and deletion in invisible. The global structure of the copies is shown on top, with arrowheads corresponding to TIRs. Region of homology to transposases sequences, as determined by BLASTX against NCBI nr protein database, or to the consensus sequence are shown in *red*. Similar copies in length and sequences defined sublineages (numbered in *green*), while a vertical *blue* line indicated a putative breakpoint that allow to divides the alignment into non-overlapping regions

The MITE_9, comprising 118 independent copies, could be unambiguously linked to the *mariner* Rpmar0 lineage, made of 8000 copies (Fig. 4a). In this case however, almost all copies had apparently different internal deletions, and were retrieved only because they have internal sequences shorter than 750 bp. The abundance of deleted copies is likely the consequence of the huge number of copies of this *mariner* lineage, but, except for few sequences, we have no trace of amplification by transposition of these shortened copies, and this cluster do not represent a *bona fide* MITE lineage, that would suggest that most of internal deletions are harmful for transposition ability. Notably, we retrieved other clusters corresponding to high copy number *mariner* lineage, but none of them actually corresponded to MITE having amplified while shortened (Table 2 and not shown).

We also could detect several clusters that are probably related to *Tc*-like or *pogo/tigger*–like elements as well as a prokaryotic IS*630* elements. The latter element could originate from endosymbiotic bacteria that are abundant and diverse in *Rhodnius* species [36]. A contamination with foreign bacterial DNA is also possible.

The MITE_109 comprised 3 sub-lineages that share similar ends but have different breakpoints. Homology with *Tigger* elements were detected in a region common to the two less abundant sub-lineages, but no longer element that could correspond to the progenitor exists in the *Rhodnius* genome (Fig. 4b).

The MITE_100 *bona fide* MITE is composed of 68 independent copies that display high homogeneity in size and sequence. The internal part of the element presents homology with *Tc1*-like elements, although we again could not find any related longer element in the genome (Fig. 4c).

MITE_120 comprised 174 independent sequences presenting homology on the main part of their external sequences. Three sub-lineages can be recognized, but concern only one third of the copies. Although clearly related, the others seemed to result from independent internal deletions from a larger element (Fig. 4d). Like for the autonomous Rpmar17 lineage, and unlike the MITE_9 previously described, it is possible to locate a potential unique breakpoint in this largest element. All deletions in the other copies are centered on this position including the 3 sub-lineages that experienced further transposition. However, this largest element is non-coding and presents no homology with any known proteins, so the autonomous partner responsible for transposition is still unidentified. Nevertheless, the TIRs sequences including a potential TA dinucleotide TSD resembles that of MITE_100 and MITE_109, suggesting that this element lineage belongs to the *Tc1*-*mariner* super-family (Table 2).

MITE_51 present a pattern similar to the MITE_120 pattern, i.e. two sub-lineages but in which most copies have suffered independent deletions, as well than a probable breakpoint at the origin of all the copies (Fig. 4e). Like for MITE_120, no homology with any proteins could be detected, the relationship with Tc1-*mariner* superfamily being only supported by the TSD and TIR sequences (Table 2).

Globally, it seems that *Tc1-mariner*, and especially *mariner* lineages are not prone to generate short MITE families. However, the fine analysis of *mariner* and the few MITE families raise interesting questions. For *mariner* the search for MITE smaller than about 800 bp was rather unfruitful. If short *mariner* MITEs exist, they are obviously in very low copy number, so not quite prone to amplification. In contrast, an important proportion of the *mariner* lineages identified correspond to shortened non-autonomous lineages usually 800–900 bp long. Altogether, this suggests than *mariner* elements are prone to deletion but the ability to transpose is likely highly constrained, by a minimum size about 800 bp, preventing the amplification or short copies and then the generation of MITE families. Noteworthy, several other *mariner* non-autonomous lineages have been detected in the drosophila genomes, most of them exhibiting a size of about 950 bp, supporting the hypothesis of a size constraint [17].

### *Dynamic of mariner transposons in the* R. prolixus *genome*

**Fig. 5:**
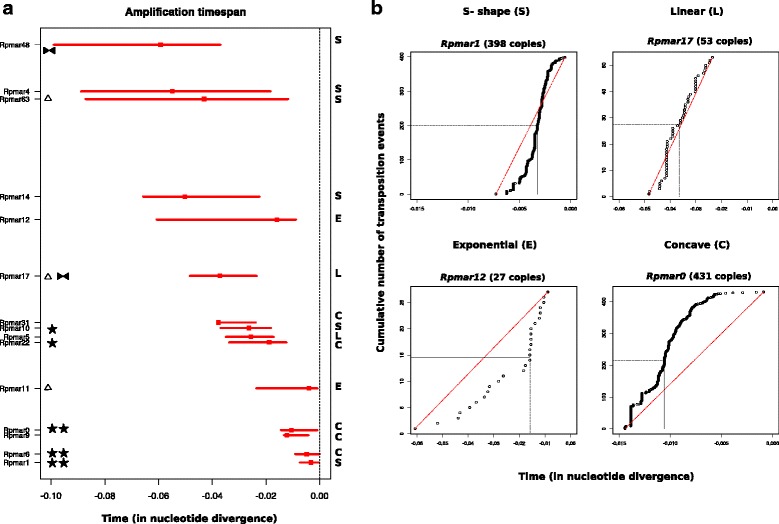
Amplification d**y**namics analyses of different *mariner* lineages found in *R. prolixus*. **a** Time-span amplification. The time span amplification is reported as a horizontal line for each lineages sorted from most recent (bottom) to most ancient (top). The position of the median transposition event is indicated by the red square. The status of each lineage appears on the left : , internally deleted lineages. , lineages with a few potentially active copies. , lineages with numerous potentially active copies.  : lineages with rearrangement. The type of dynamics is reported on the right (S: S-shaed, L: Linear, C: Concave, E: Exponential). **b**. Examples of amplification dynamics**.** Each curve represents a Lineage-Through-Time (LTT) plot i.e. the cumulative number of transposition events over time, measured in genetic divergence units (from present). The red dotted line represents the theoretical curve obtained for a constant rate of transposition over time. The position of the median transposition events is indicated by horizontal and vertical dotted lines. Given the very high copy number of Rpmar0, we present a plot with a random sampling of 431 copies

We used the methodology developed by Le Rouzic et al. [21] to infer the dynamics and activity of *mariner* families identified in the *R. prolixus* genome*.* Based on the phylogenetic relationships of the sequences present in one species, it avoids the bias of pairwise or consensus comparisons and provides an estimation of the amplification time-span of a lineage, as well as the variation of the duplicative transposition rate. We analyzed a representative set of 15 *mariner* families. The time-span of each lineage is reported on Fig. 5a, with an indication of the time at which half of the transposition events have occurred. Although the time-span may be overestimated for older lineages, the comparison of the 15 lineages suggests that at any time several mariner lineages have been active at the same time. Among the five more recent lineages, four seem to be still active (transposition events at time 0), which is expected for three of them that are characterized by numerous potentially active copies (2 stars). Although recently active, the Rpmar9 lineage does not seem to transpose anymore, which is in accordance with the absence of intact copies. More curiously, Rpmar11 is still active, although this lineage consists only of internally deleted copies. Recent transposition of this lineage likely occurs using the transposase of another active lineage, such as Rpmar6, that shares almost the same TIRs as Rpmar11. Among older lineages, only two still contain a few copies with uninterrupted ORF, but obviously, the lineages are now extinct. The position of the median transposition event greatly varies depending of the lineage and reflects the existence of four different dynamics that are exemplified in Fig. 5b.

The first pattern is a “S-shaped” curve, which reflects the fact that transposition started with a very slow rate, then the rate increased before slowing down progressively. This pattern can be interpreted as a transposition rate that is dependent of copy number at the beginning. At the end of the amplification period, the slowing down may be due to the progressive loss of active copy (inactivation), or the establishment of regulations. In such a dynamics, the median transposition event is roughly located at mid-course of the amplification time-span. This dynamics is observed for older lineages but also in recent ones, such as Rpmar1.

A second type of dynamics is referred as “Exponential”, and is compatible with a model in which the transposition rate per copy is constant, meaning the more copies the more transposition events. This is expected for the beginning of the amplification (before establishment of regulations), or for active lineages (undergoing amplification), for example in *Rpmar11. Rpmar12* also display this dynamics, although it is now inactive, which indicates that the transposition suddenly stopped after the initial transposition burst, maybe due by the rapid loss of all active copies. The median is then shifted to the recent time.

The third dynamics is described as “Linear”, because the transposition rate seems to be constant over time, until it falls rapidly to zero. In this case, it is independent of the copy number. *Rpmar17* and *Rpmar5* follow this dynamics, characterized by a median centered in the middle of the amplification time-span.

Finally the fourth dynamics is called “Concave” and is characterized by a high transposition rate at the beginning, followed by a progressive slowing down. The median is the shifted to ancient time, and several recent or middle-aged lineages present this dynamics.

This comparative analysis revealed that very different dynamics characterize closely related TE lineages that coexist in the same genome at the same time. These differences can be explained by the intrinsic biochemical properties of the element [37], or the establishment of specific regulations, through epigenetic silencing, or through cell cycle-coupled controls [38–40]. It should be noticed that methodological biases exist since the method relies on a reconstructed phylogeny based on extant copies, and only duplicative transposition are scored. The resulting dynamics can also be modified by variable deletion rates. However, considering that the same genomic deletion rate will apply for coexisting lineages, we suspect that it cannot be responsible for the dynamics differences we observed between lineages.

Globally, it appears that the *R. prolixus* genome is recurrently and frequently invaded by *mariner* elements. *Mariner* elements seem to escape easily transposition controls since huge copy number are observed for several lineages. In particular the three most abundant *mariner* lineages (8,041, 767 and 488 total copies) are also the most recent ones. The high level of amplification is not compensated by a high turnover, i.e., the rapid disappearance of older lineages, as shown by the large diversity of *mariner* lineages and the high copy number in old lineages too.

### Evidence of multiple HT of Mariner elements

We screened the complete GenBank and WGS databases with the *mariner* and MITE *mariner* consensus in order to document the propensity of *mariner* TEs in *R. prolixus* to generate HTs.

We detected ten putative cases of HT (Table 3). One case concerns a MITE element found in the genome of the flatworm *Schmidtea mediterranea,* a free-living freshwater planarian that preys on small arthropods and gastropods. Despite a time divergence of about 792 Ma between this species and *R. prolixus,* the level of similarity between the two elements is 97 % (http://www.timetree.org/index.php)[41]. Interestingly, in *S. mediterranea,* sequences homologous to MITE_185 of *R. prolixus* correspond to a complete *mariner* element present in numerous copies. This would indicate that MITE_185 is an internal, deleted derivative of an autonomous transposon still present in *S. mediterranea*. Surprisingly, the flatworm complete *mariner* element is lacking in the genome of *R. prolixus.* This situation is a reminiscence of the *hAT* MITEs found in *R. prolixus* that were horizontally transferred with diverse mammals [2]. A direct TE acquisition from a flatworm by the terrestrial haematophagous *R. prolixus* seems unlikely. The more plausible scenario is a transfer in the *R. prolixus* genome, from an unidentified source, of the complete element followed by a deletion event that gave rise to MITE_185 element and a secondary lost of the complete element. Subsequently, they have expanded through cross mobilization with different, non-homologous, *mariner* elements already present in the bug genome. Alternatively, the autonomous element that has generated the *Rhodnius* MITE has been lost after the MITE amplification.

**Table 3:** List of the different HTs of *mariner* elements found in the *R. prolixus* genome

*Rhodnius* TE	First BLAST Hit	Copy number	Nucleotide similarity
MITE_185	*Schmidtea mediterranea* (flatworm)	79	97 %
Rpmar0	*Dendroctonus ponderosae* (beetle)	38	87 %
Rpmar5	*Hymenolepis diminuta* (tapeworm)	205	93 %
Rpmar10	*Haemonchus placei* (nematode)	77	75 %
Rpmar12	*Scolia oculata* (parasitic wasp)	4	87 %
Rpmar12	*Bombus terrestris* (bumblebee)	17	93 %
Rpmar27	*Glossina pallipes* (tsetse fly)	1	81 %
Rpmar30	*Artibeus jamaicensis* (bat)	3	83 %
Rpmar57	*Drosophila* species	>30	91 %
Rpmar63	*Strongyloides stercoralis* (nematode)	2	83 %

The nine other putative cases of HT concerned *mariner* autonomous transposons. They involved five other insects (*Dendroctonus ponderosae*, *Scolia oculata*, *Bombus terrestris*, *Glossina pallipes*, and *Drosophila sp*.), one south American bat (*Artibeus jamaicensis*), two blood sucker nematodes of mammals (*Haemonchus placei* and *Strongyloides stercoralis)* and one tapeworm *(Hymenolepis diminuta*) that parasites various insects and mammals. Phylogenetic analysis of the transposase of each element confirms the close proximity between these elements and the *R. prolixus* transposons (Fig. 2). Concerning putative TE transfer between *R. prolixus* and the four other insects, despite high level of sequence conservation between these elements and the *R. prolixus mariner* transposons (up to 93 %), the long period of divergence since the split between Hemiptera and Diptera/Hymenoptera (>300 Ma [41]) is incompatible with a vertical inheritance. For horizontal transfers two scenario of transmission could be examined: direct transmission or indirect *via* intermediate hosts. Interestingly, the implication of parasitoid insects as vector of HT of *hAT* and *Ginger* MITEs between *R. prolixus* and the silkworm *B. mori* has been proposed [10]. A similar situation has been reported between *R. prolixus* and the twisted wing parasite *Mengenilla moldrzyki* that are known to infect a large variety of insects [42]. In our dataset, we have detected a possible HT between the parasitic wasp *S. oculata* and *R. prolixus*. Eggs of Triatomine bugs as *Rhodnius* species are effectively infected by diverse parasitic wasps [43]. Another example of HT of a *mariner* element between the parasitic wasp *Ascogaster reticulatus* and its host the moth *Adoxyphyes honmai* has also been evidenced [44]. Since the implication of insect parasites as intermediate vectors seems to be plausible, this mechanism could be considered for the sharing of very closely related *mariner* transposons between *R. prolixus* and *Drosophila sp.*, *B. terrestris* and *G. pallipes*. In the case of the parasite tapeworm *H. diminuta,* both the strong transposon sequence conservation between this tapeworn and *R. prolixus* and the ecology of this organism that live as a parasite of various insects, are arguments in favor a recent and direct HT within *R. prolixus*. Moreover, a direct HT between the South American bat *A. jamaicensis* and *R. prolixus* is possible, since *R. prolixus* is known to feed on bats blood [45]. Interestingly, HTs of *mariner* elements have been evidenced between various insects and mammals [46]. Concerning the two species of blood feeding nematodes of mammals, as *R. prolixus* infects the same range of hosts, we cannot ruled out the hypothesis of independent HTs of the transposons from a common but unknown mammalian host.

Taken together our data indicate the existence of frequent HTs of *mariner* transposons between *R. prolixus* and a large variety of organisms. In addition, keeping in mind the recent data supporting the existence of other transposon HTs with mammals [2, 47] and insects [10, 48], our analyses demonstrate the existence of a diverse horizontal flux of transposons in the genome of *R. prolixus*. By providing new invading elements, we can hypothesize that this flux balances the inevitable stochastic losses of m*ariner* elements and thus participate to the strong preponderance of this super-families in the *R. prolixus* genome.

## Conclusion

Combining library-based and *de-novo* methods of TEs detection in the *R. prolixus* genome, we showed that *mariner* transposons outnumbered the other super-families representing 75 % of the mobilome. Compared to other insect genomes, this unusual dominance of *mariner* elements could be explained by at least three factors acting in concert:

i) a long-lasting permissibility of the genome for *mariner* that leads to lineages with huge copy number. Copy number explosion is especially striking for recent still active *mariner* lineages, but is also observed in very old lineages supposed to progressively loose copies. A recent burst of a single *mariner* lineage has led to the generation of more than 8000 copies (two third of the total *mariner* elements present in the genome);
ii) a huge diversity of *mariner* lineages that was never observed before since between 32 and 89 different lineages were recovered. These lineages are usually well delimited and reflect the diversity of *mariner* within the whole metazoan clade, since lineages from most *mariner* subfamilies could be identified.
iii) frequent occurrence of HT of *mariner* elements within various species including other insects in particular parasitoids, hematophagous nematodes, parasite worms and a South American bat.

Finally, this huge dataset of copies has revealed some aspect of the biology of *mariner* elements, for example, the generation of shorter lineages that seems to be highly constrained by size, the fact that these shorter lineages are frequent within some subfamilies only, that rearranged lineages can also arise by 5’ replacement with 3’part. We believe that the data and interpretations provided here will offer a basis to future study aiming to understand the role play by transposable elements during evolution and the adaptation to human of Triatomine bugs.

## Additional files

Additional file 1: Table S1. Transposases sequences used as queries in the TBLASTN search (GI, family and subfamily) with the number of clusters obtained by a reciprocal BLASTX using the longest element of each cluster (PDF 30 kb) Additional file 2: Figure S2. A. Histograms showing the positions of the breakpoints in copies showing a rearranged structure. Copies were retrieved after megablastn using the full-length sequence as query, and were reversed-blasted against the full-length sequence. Only copies displaying hits on both strands were kept. B. Plot density illustrating the nonrandom distribution of breakpoints in A and B parts together). C. Scatter plot showing breakpoints in A part vs B part for each copy. (PDF 84.9 kb)
