# Supplementary material for: Mariner transposons are sailing in the genome of the blood-sucking bug Rhodnius prolixus
Source: BMC Genomics. 2015 Dec 15;16:1061. doi: 10.1186/s12864-015-2060-9 (PMC4678618; doi:10.1186/s12864-015-2060-9)
Supplement: Additional file 1: Table S1. — Transposases sequences used as queries in the TBLASTN search (GI, family and subfamily) with the number of clusters obtained by a reciprocal BLASTX using the longest element of each cluster (PDF 30 kb) [file 12864_2015_2060_MOESM1_ESM.pdf]

Table S1.

Transposases sequences used as queries in the TBLASTN search (GI, family and subfamily) with the number of clusters obtained by a reciprocal BLASTX using the longest element of each cluster.

Transposase	Accession (GI) or reference	Family based on catalytic domain	Subfamily	Number of clusters
		mariner search		
MOS1	75009976	*mariner* (DD(34)D	mauritiana	12
HVMAR1	1708663	*mariner* (DD(34)D		2
DGMAR1	887424	*mariner* (DD(34)D	cecropia	3
CEMAR1	7331903	*mariner* (DD(34)D	elegans	0
FAMAR1	75008822	*mariner* (DD(34)D	mellifera	22
PACMAR2	19692999	*mariner* (DD(34)D	marmorata	1
DROMAR23	Wallau *et al.* 2014	*mariner* (DD(34)D	irritans	17
DROMAR8	Wallau *et al.* 2014	*mariner* (DD(34)D	drosophila	32
		Non-mariner search		
Tc1	141446	*Tc1* (DD34)E		8
Tc3	464866	*Tc1* (DD34)E		4
S	2133730	*Tc1* (DD34)E		20
Ag	16266047	DD(37)E		0
Tc4	156453	*Pogo* (DDxD)		
Jerky	157822169	*Pogo* (DDxD)		7
Tigger1	2226004	*Pogo* (DDxD)		3
PogoR11	2133672	*Pogo* (DDxD)		
Lemi1	4262216	*Pogo* (DDxD)		
Fot1	2723	*Pogo* (DDxD)		
Fot5	38520869	*Pogo* (DDxD)		2
Bmmar1	1698453	DD(37)D		7
Ant1	191148	DDxE		1
Offalax	438828	DDxE		
rhizobium	1072027	DDxE		2
