# Supplementary material for: Mariner transposons are sailing in the genome of the blood-sucking bug Rhodnius prolixus
Source: BMC Genomics. 2015 Dec 15;16:1061. doi: 10.1186/s12864-015-2060-9 (PMC4678618; doi:10.1186/s12864-015-2060-9)

Figure S2 : **A.** Histograms showing the positions of the breakpoints in copies showing a rearranged structure. Copies were retrieved after megablastn using the full-length sequence as query, and were reversed-blasted against the full-length sequence. Only copies displaying hits on both strands were kept.
B. Plot density illustrating the nonrandom distribution of breakpoints in A and B parts together).
C. Scatter plot showing breakpoints in A part vs B part for each copy.

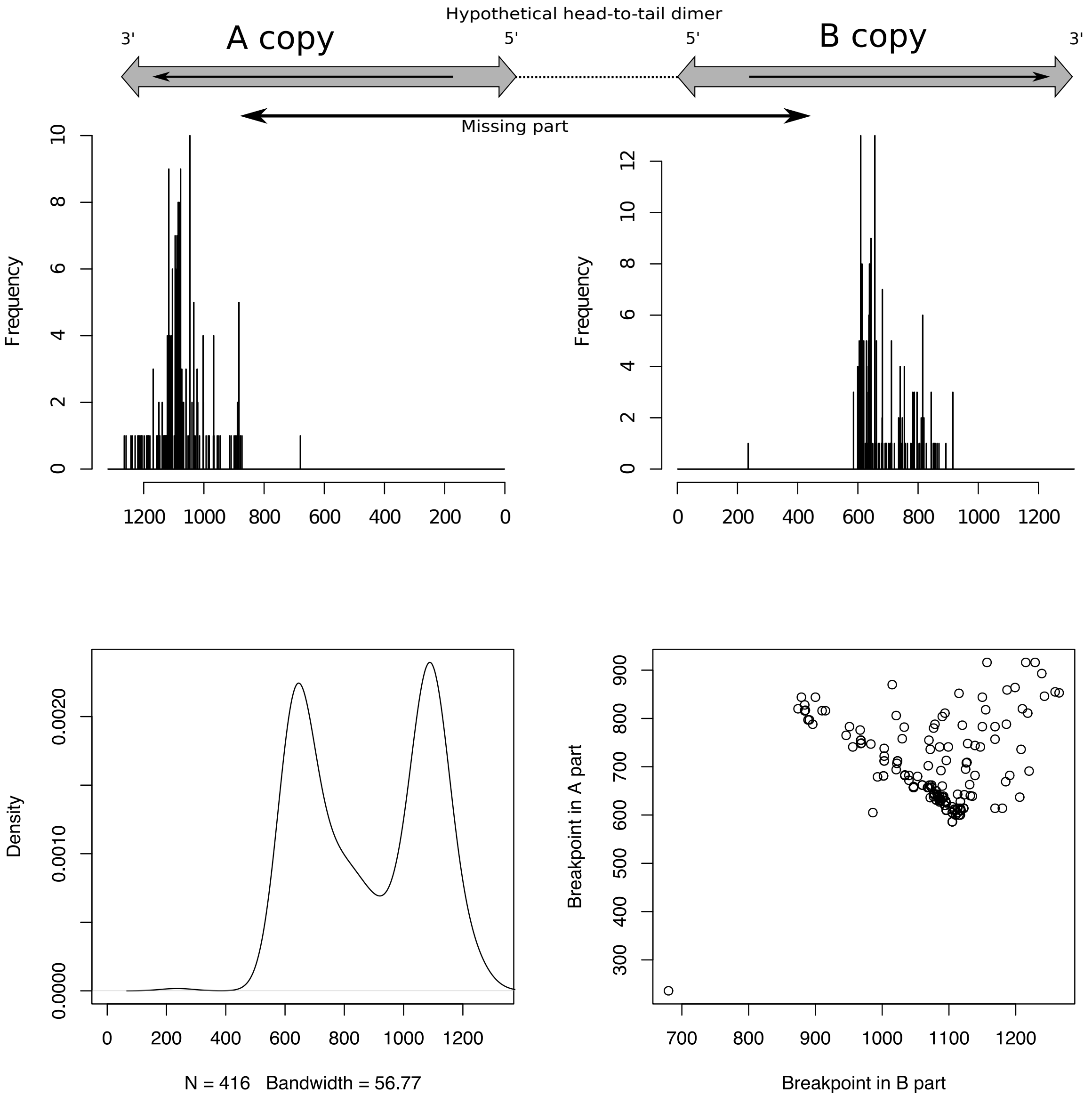

Supplement: Additional file 2: Figure S2. — A. Histograms showing the positions of the breakpoints in copies showing a rearranged structure. Copies were retrieved after megablastn using the full-length sequence as query, and were reversed-blasted against the full-length sequence. Only copies displaying hits on both strands were kept. B. Plot density illustrating the nonrandom distribution of breakpoints in A and B parts together). C. Scatter plot showing breakpoints in A part vs B part for each copy. (PDF 84.9 kb) [file 12864_2015_2060_MOESM2_ESM.pdf]
